# *Rickettsia felis* Infection in Febrile Patients, Western Kenya, 2007–2010

**DOI:** 10.3201/eid1802.111372

**Published:** 2012-02

**Authors:** Alice N. Maina, Darryn L. Knobel, Ju Jiang, Jo Halliday, Daniel R. Feikin, Sarah Cleaveland, Zipporah Ng’ang’a, Muthoni Junghae, Robert F. Breiman, Allen L. Richards, M. Kariuki Njenga

**Affiliations:** Author affiliations: Jomo Kenyatta University of Agriculture and Technology, Nairobi, Kenya (A.N. Maina, Z. Ng’ang’a);; Kenya Medical Research Institute, Kisumu, Kenya (A.N. Maina, D.L. Knobel),; University of Pretoria, Onderstepoort, South Africa (D.L. Knobel);; Naval Medical Research Center, Silver Spring, Maryland, USA (A.L. Richards, J. Jiang);; University of Glasgow, Glasgow, Scotland, UK (J. Halliday, S. Cleaveland);; US Centers for Disease Control and Prevention, Nairobi (M. Junghae, D.R. Feikin, R.F. Breiman, M.K. Njenga)

**Keywords:** rickettsial infections, *Rickettsia felis*, rickettsia, Kenya, fever of unknown origin

## Abstract

To determine previous exposure and incidence of rickettsial infections in western Kenya during 2007–2010, we conducted hospital-based surveillance. Antibodies against rickettsiae were detected in 57.4% of previously collected serum samples. In a 2008–2010 prospective study, *Rickettsia felis* DNA was 2.2× more likely to be detected in febrile than in afebrile persons.

Rickettsioses are a major human health problem in many parts of the world, including sub-Saharan Africa (*1**,**2*). Awareness of rickettsiae as causes of public health problems has been increasing; several novel or emerging diseases caused by these pathogens have been recognized. In Kenya, recent reports have documented human infections with *Rickettsia conorii* (*3**,**4*) and *R. felis* (*5*) and tick infection with *R. africae* (*6*). Our objectives were to assess previous human exposure to rickettsiae and to determine the incidence of rickettsial infections among febrile and afebrile persons in western Kenya.

## The Study

**Figure 1:**
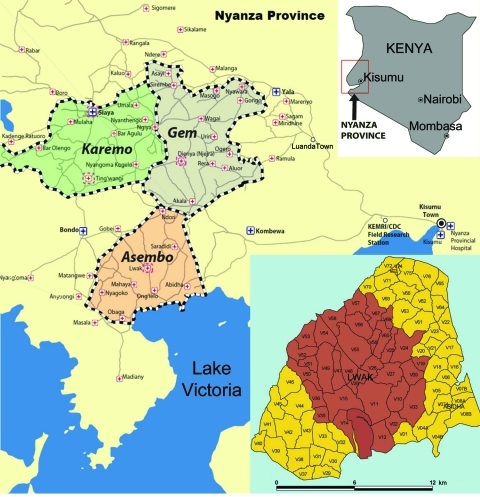
Locations of villages (brown shading in inset map) in Asembo area of western Kenya where the study was conducted, January 2007 through October 2008. Used with permission of Kenya Medical Research Institute/Centers for Disease Control and Prevention Research and Public Health Collaboration.

The study was conducted among patients visiting the Lwak Mission Hospital, a rural health care facility in western Kenya in the Asembo area, Rarieda District, in western Kenya (Figure 1). Lwak Mission Hospital serves as the field clinic for population-based infectious disease surveillance conducted by the Kenya Medical Research Institute and the US Centers for Disease Control and Prevention as described (*7*). The study was conducted with ethical approval from these institutions (protocol nos. 932 and 4566, respectively). To assess previous exposure to rickettsiae, we examined a randomly selected subset of 357 serum specimens collected January 2007 through October 2008 from patients participating in population-based infectious disease surveillance. Samples were screened at a dilution of 1:128 for IgG against spotted fever group (SFG) and typhus group (TG) rickettsiae by using an indirect fluorescence antibody assay (Fuller Laboratories, Fullerton, CA, USA).

Blood specimens were collected from the first 2 outpatients ≥5 years of age and the first 2 outpatients <5 years of age seen each day for acute febrile illness (recorded axillary temperature >38.0°C without an obvious cause, defined as cough, difficulty breathing, chest pain, signs of meningitis, or bloody diarrhea) from November 2008 through February 2010. A positive malaria smear was not an exclusion criterion. During the same period, blood specimens were also collected from controls: a group of outpatients who did not have febrile, respiratory, or diarrheal illness during the preceding 2 weeks and asymptomatic persons who accompanied patients to the clinic.

To detect rickettsial DNA, we performed 3 quantitative PCRs: a genus-specific assay selective for a 74-bp segment of the citrate synthase (*gltA*) gene, a group-specific assay that detects a 128-bp segment of the outer membrane protein (*ompB*) gene for tick-borne rickettsiae, and a species-specific assay that detects a 129-bp segment of the *ompB* gene for *R. felis* (*5**,**8*). To identify which *Rickettsia* sp. was present in the positive specimens, we PCR amplified and sequenced segments of 4 rickettsial genes—*17-kDa*, *ompB*, and 2 *R. felis* plasmid genes (*pRF* and *pRFδ*)—by using primers and procedures as described (*5*).

**Figure 2:**
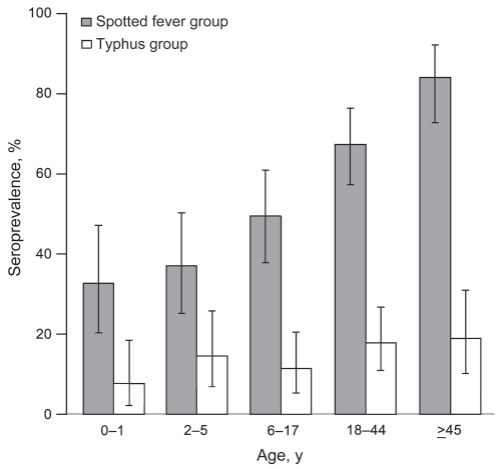
Age-stratified seroprevalence of IgG to rickettsiae among patients participating in population-based infectious disease surveillance, January 2007 through October 2008. Vertical lines indicate 95% binomial CIs.

Overall, 205 (57.4%) of 357 specimens had antibodies against rickettsiae. Of 357 serum specimens tested, 200 (56.0%, 95% exact binomial CI 50.7%–61.2%) had detectable IgG against SFG antigen preparation. Antibodies against TG antigen preparation were detected in 52 (14.5%, 95% CI 11.0%–18.6%) of 357 specimens tested; 47 (90.4%) of these specimens that reacted to TG antigens were also positive for SFG antigens, and 5 (1.4%) of the 357 specimens were positive for TG antigens alone. Presence of antibodies against SFG or TG antigens was not associated with patient sex (p>0.05). In addition, patient age was not significantly associated with TG seropositivity (χ^2^ for linear trend 3.41, df 1, p = 0.065). However, an incremental linear association was demonstrated between age and IgG seropositivity to SFG (χ^2^ for linear trend 45.46, df 1, p<0.001) (Figure 2).

A total of 699 febrile patients who sought care at Lwak Mission Hospital from November 2008 through February 2010 and 236 afebrile persons enrolled during this same period were tested for rickettsiae (Table 1). Overall, 50 (7.2%, 95% CI 5.4%–9.3%) of the febrile patients and 8 (3.4%, 95% CI 1.5%*–*6.6%) of the afebrile persons had positive rickettsiae results according to the genus-specific *gltA* assay. Univariate logistic regression indicated that febrile patients were more likely than afebrile persons to have positive PCR results (odds ratio 2.20, 95% CI 1.03–4.70, p = 0.04). According to the *ompB* assay, all specimens tested were negative for tick-borne rickettsiae. BLAST searches (www.ncbi.nlm.nih.gov/blast/Blast.cgi) for homologous sequences determined that the segments amplified from the 3 genes had 100% nt homology with *R. felis* URRXWCal2 (Table 2).

**Table 1:** Demographic characteristics of participants in hospital-based survey, western Kenya, 2008–2010

Characteristic	No. (%) febrile, n = 699	No. (%) afebrile, n = 236	p value*
Age group, y			<0.001
0–1	61 (8.7)	15 (7.0)	
2–5	345 (49.4)	30 (14.1)	
6–17	214 (30.6)	63 (29.6)	
18–44	61 (8.7)	72 (33.8)	
>45	18 (2.6)	33 (15.5)	
Missing	0	23	
Sex			<0.001
M	352 (50.6)	73 (30.4)	
F	344 (49.4)	163 (69.1)	
Missing	3	0	

*By χ^2^ test.

**Table 2:** Genetic sequence analysis results of rickettsial DNA amplified from *17-kDa*, *ompB*, and *pRF* genes from 21 human specimens, Lwak Mission Hospital, Western Kenya, 2008–2010*

DNA no.	*17-kDa* gene sequence	*ompB* gene sequence†	*pRF* gene sequence	*pRFδ* gene sequence
1	+	+	–	–
2	+	+	+	–
3	+	+	–	–
4	+	+	–	–
5	+	+	–	–
6	+	+	–	–
7	+	+	–	–
8	+	+	–	–
9	+	–	–	–
10	+	+	+	–
11	+	–	–	–
12	+	–	–	–
13	+	–	–	–
14	+	+	–	–
15	+	+	+	–
16	+	+	–	–
17	+	–	–	–
18	+	+	+	–
19	+	+	+	–
20	+	–	+	–
21	+	+	+	–

*omp, outer membrane protein; pRf, *R. felis* plasmid; +, positive; –, negative. †*Rickettsia* DNA from fleas, dogs, and cats had 93%–99% nt sequence homology with *R. felis.*

In addition to fever, the most common clinical manifestations among patients with positive PCR results for rickettttsiae were headaches (100%), chills (93.8%), muscle aches (68.8%), and joint pains (68.8%). Rash was reported for 4.4% of rickettsiae-positive patients. Among febrile patients, no statistically significant associations were found between specific signs or symptoms and positive PCR results for rickettsiae (p>0.05). Samples from all febrile patients were Giemsa stained and examined; malaria parasites were detected in 79.2% and 73.4% of samples from patients who had PCR-positive and PCR-negative results for rickettsiae, respectively.

## Conclusions

The 2007–2008 serosurvey found prevalence of IgG against rickettsiae to be high. Other countries in Africa have reported similar (28%–58%) seroprevalence (*9**,**10*). The finding that prevalence of IgG to SFG rickettsiae increased with age can, in part, be explained by cumulative exposure to the pathogen and lifelong persistence of IgG. The high number of patients seropositive for SFG and TG rickettsiae may be attributed to cross-reactivity between SFG and TG rickettsial antigens (*11*), although results from studies in animal models show that cross-reactivity between SFG and TG rickettsiae is not consistent (*12*). Znazen et al. (*13*) speculate that antibodies against *R. felis* may be the major cause of cross-reactions to TG-rickettsiae–specific and SFG-rickettsiae–specific antigens.

The identification of *R. felis* DNA sequences in febrile patients confirms the previous finding of this pathogen among febrile patients in Kenya (*5*). Our findings are also similar to those from northern Tanzania, where acute rickettsiosis was serologically confirmed for 8% of febrile hospital inpatients (*14*), and from rural Senegal, where 6% of febrile patients without malaria had positive *R. felis* test results (*15*). Our findings suggest that rickettsial infections should be considered in the differential diagnosis of febrile cases in western Kenya and that diagnostic capacity should be established. Clinicians in malaria-endemic areas should consider rickettsial co-infections in diagnostic protocols and treatment of patients with malaria and other febrile illnesses.
